# Supplementary material for: Diffusion Tensor and Dynamic Contrast-Enhanced Magnetic Resonance Imaging Correlate with Molecular Markers of Inflammation in the Synovium
Source: Diagnostics (Basel). 2022 Dec 5;12(12):3041. doi: 10.3390/diagnostics12123041 (PMC9776499; doi:10.3390/diagnostics12123041)
Supplement: Supplementary file 1 [file diagnostics-12-03041-s001.zip › diagnostics-1994743-supplementary.pdf]

**Table S1: Correlation between different DTI and DCE parameters and different severities of inflammation based on Rooney synovitis score**

| S.No. | | Mild inflammation (Rooney score 0-1.5) n=16 | | Moderate inflammation (Rooney score 1.6-3.0) n=27 | | Severe Inflammation (Rooney score 3.1-5.0) n=37 | |
|---|---|---|---|---|---|---|---|
| | Parameter | correlation | significance | correlation | significance | correlation | significance |
| 1 | FA | .518 | .024 | .764 | .000* | .927 | .000* |
| 2 | MD | -.505 | .027 | -.255 | .095 | -.435 | .004* |
| 3 | Kep | .042 | .441 | -.004 | .493 | .571 | .000* |
| 4 | BF | -.127 | .326 | .408 | .016 | .594 | .000* |
| 5 | CL | .155 | .291 | .075 | .353 | .638 | .000* |
| 6 | CP | .138 | .311 | .083 | .337 | .260 | .063 |
| 7 | CS | -.159 | .285 | -.055 | .391 | -.397 | .008 |
| 8 | BV | .113 | .345 | .311 | .054 | .519 | .001* |

*Correlation is significant at $p<0.005$ (as per Bonferroni correction)

FA: Fractional Anisotropy, MD: Mean Diffusivity, CL: Linear anisotropy, CP: Planar anisotropy, CS: Spherical isotropy, BV: Blood Volume, BF: Blood Flow, k ep: Volume transfer constant

**Table S2. Correlation between the values of DTI & DCE –MRI indices with various infiltrating immune cells in the synovium of different group of arthritis patients (n=80). Results are expressed as R values.**

| **RA (n=6)** | **Infiltrated Immune cells in synovium** | | | | | | | | | | |
|---|---|---|---|---|---|---|---|---|---|---|---|
| **DTI & DCE-MRI Indices** | CD3 | CD 4 | CD 8 | CD 20 | CD34 | CD 68 | CD 138 | CD 54 | TNF-α | IL-1β | Total Inflammatory cells |
| FA | 0.38* | 0.48* | 0.67** | 0.39* | 0.42* | 0.42* | 0.80* | 0.46** | 0.23 | 0.48* | 0.72** |
| MD | 0.01 | -0.50* | 0.04 | -0.58* | -0.34 | 0.46 | -0.32* | 0.36 | 0.27 | -0.08 | -0.40* |
| CL | 0.55* | 0.02 | 0.40 | 0.28 | 0.32 | 0.08 | 0.83* | 0.24 | 0.13 | 0.02 | 0.08 |
| CP | 0.38 | 0.78 | 0.15 | 0.58 | 0.21 | 0.03 | 0.45 | 0.55 | 0.48 | 0.03 | 0.19 |
| CS | 0.07 | -0.46* | -0.42 | -0.79 | 0.22 | -0.33 | 0.76 | 0.76 | -0.57 | 0.27 | 0.44 |
| BF | 0.32 | 0.22 | 0.52* | 0.34 | 0.85* | 0.32 | 0.44* | 0.42 | 0.23 | 0.32 | 0.53* |
| BV | 0.21 | 0.53 | 0.32 | 0.33 | 0.85* | 0.42 | 0.41 | 0.12 | 0.22 | 0.54* | 0.47* |
| $k_{ep}$ | 0.12 | 0.23 | 0.53 | 0.42 | 0.77* | 0.32 | 0.31 | 0.42 | 0.43* | 0.32* | 0.43 |
| PCI | 0.56 | 0.23 | -0.01 | 0.14 | -0.28 | 0.46 | 0.55 | 0.66 | 0.35 | 0.68 | 0.68 |
| **Infective Arthritis (n=19)** | | | | | | | | | | | |
| FA | 0.56* | 0.51* | 0.88* | 0.30 | 0.45* | 0.70* | 0.33 | 0.22 | 0.31 | 0.78* | 0.86** |

| | | | | | | | | | | | |
|---|---|---|---|---|---|---|---|---|---|---|---|
| MD | -0.52* | -0.12 | -0.63* | 0.12 | -0.43 | 0.10 | -0.13 | 0.03 | 0.08 | 0.76* | -0.58* |
| CL | 0.62* | 0.32 | 0.42 | 0.33 | 0.21 | -0.36 | 0.40 | 0.16 | 0.09 | 0.57 | 0.75* |
| CP | 0.16 | -0.27 | -0.15 | 0.31 | 0.11 | 0.23 | 0.19 | -0.12 | -0.17 | 0.11 | 0.17* |
| CS | -0.12 | 0.15 | -0.08 | 0.05 | 0.23 | -0.13 | -0.20 | -0.19 | -0.05 | -0.21 | -0.25 |
| BF | 0.32 | 0.12 | 0.34 | 0.11 | 0.89** | 0.23 | 0.34 | 0.11 | 0.41 | 0.12 | 0.45* |
| BV | 0.41 | 0.32 | 0.12 | 0.18 | 0.93** | 0.14 | 0.42 | 0.17 | 0.31 | 0.15 | 0.49* |
| k ep | 0.31 | 0.33 | 0.15 | 0.17 | 0.27 | 0.24 | 0.32 | 0.12 | 0.01 | 0.33 | 0.34 |
| PCI | -0.33 | -0.13 | -0.14 | -0.14 | -0.08 | -0.35 | 0.09 | -0.03 | -0.1 | -0.28 | -0.48 |
| **USpA (n=14)** | | | | | | | | | | | |
| FA | 0.68* | 0.58* | 0.75* | -0.07 | 0.43* | 0.73* | 0.16 | 0.51* | 0.30 | 0.10 | 0.81** |
| MD | -0.58* | -0.29 | -0.45* | 0.23 | -0.45 | -0.42* | 0.08 | -0.14 | 0.05 | -0.16 | -0.63* |
| CL | 0.57* | 0.49 | 0.00 | 0.54* | 0.12 | 0.49* | 0.34 | 0.14 | 0.14 | 0.17 | 0.14 |
| CP | 0.45 | 0.23 | 0.28 | 0.48 | 0.14 | 0.27 | 0.14 | 0.23 | 0.47 | 0.08 | 0.36 |
| CS | -0.27 | -0.40 | -0.29 | 0.54* | 0.21 | -0.56* | 0.57* | -0.15 | -0.61* | -0.39 | -0.23 |
| BF | 0.45* | 0.32 | 0.45 | 0.44 | 0.82** | 0.42 | 0.24 | 0.34 | 0.54* | 0.11 | 0.34* |
| BV | 0.31 | 0.43 | 0.56* | 0.44 | 0.85** | 0.35 | 0.335 | 0.12 | 0.34 | 0.24 | 0.45* |

| | | | | | | | | | | |
|---|---|---|---|---|---|---|---|---|---|---|
| k ep | 0.11 | 0.23 | 0.24 | 0.15 | 0.34 | 0.46 | 0.32 | 0.21 | 0.33 | 0.25 | 0.25 |
| PCI | -0.40 | -0.06 | -0.11 | 0.12 | 0.01 | -0.30 | -0.07 | -0.67** | -0.10 | 0.23 | -0.43 |
| **UCMA (n=31)** | | | | | | | | | | | |
| FA | 0.64* | 0.76* | 0.31 | 0.27 | 0.22 | 0.30 | 0.16 | 0.33 | 0.11 | 0.08 | 0.77** |
| MD | -0.51* | -0.16 | -0.16 | -0.48* | 0.31 | 0.29 | 0.05 | 0.00 | 0.19 | 0.00 | -0.49* |
| CL | 0.36 | -0.09 | -0.12 | -0.04 | 0.35 | 0.29 | -0.07 | 0.04 | 0.18 | 0.24 | 0.27 |
| CP | -0.25 | 0.05 | -0.03 | 0.13 | 0.17 | -0.43 | 0.11 | 0.14 | -0.23 | -0.43 | -0.21 |
| CS | 0.30 | -0.01 | 0.39 | 0.11 | 0.11 | 0.25 | 0.62** | 0.37 | 0.29 | 0.01 | 0.55* |
| BF | 0.35 | 0.46* | 0.51* | 0.32 | 0.66** | 0.33 | 0.38 | 0.35 | 0.11 | 0.14 | 0.64* |
| BV | 0.37* | 0.44* | 0.53* | 0.54* | 0.70** | 0.13 | 0.17 | 0.06 | 0.42 | 0.29 | 0.56* |
| k ep | 0.23 | 0.15 | 0.22 | 0.15 | 0.35 | 0.15 | 0.15 | 0.07 | 0.54 | 0.35 | 0.46 |
| PCI | -0.40 | 0.06 | -0.11 | 0.12 | 0.50 | -0.30 | 0.07 | -0.67** | 0.23 | -0.10 | -0.43 |
| **OA (n=4)** | | | | | | | | | | | |
| FA | 0.42 | 0.53* | 0.43 | 0.75 | 0.34 | 0.78** | 0.72** | 0.75* | 0.55 | 0.82 | 0.84** |
| MD | -0.58 | -0.68 | -0.93 | 0.02 | 0.44* | -0.27 | -0.66 | 0.05 | -0.44 | 0.15 | -0.61* |
| CL | 0.55* | 0.02 | 0.70* | 0.28 | 0.23 | 0.08 | 0.83* | 0.24 | 0.08 | 0.23 | 0.08 |
| CP | 0.38 | -0.78 | 0.15 | 0.58 | 0.15 | -0.03 | -0.45 | 0.55 | 0.48 | 0.03 | 0.19 |

| | | | | | | | | | | | |
|---|---|---|---|---|---|---|---|---|---|---|---|
| CS | 0.07 | -0.46* | 0.42 | 0.28 | 0.19 | 0.33 | 0.76 | 0.76 | 0.57 | 0.27 | 0.44 |
| BF | 0.24 | 0.56 | 0.22 | 0.35 | 0.68* | 0.31 | 0.18 | 0.26 | 0.33 | 0.17 | 0.25 |
| BV | 0.15 | 0.31 | 0.15 | 0.23 | 0.86* | 0.21 | 0.22 | 0.42 | 0.24 | 0.27 | 0.46 |
| k ep | 0.04 | 0.04 | 0.13 | 0.22 | 0.13 | 0.22 | 0.16 | 0.31 | 0.17 | 0.15 | 0.11 |
| PCI | -0.61 | 0.19 | 0.61 | -0.61 | 0.48 | -0.54 | -0.12 | -0.64 | -0.88 | -085 | -0.81 |
| **Others (n=6)** | | | | | | | | | | | |
| FA | 0.60* | 0.85** | 0.41 | 0.00 | 0.24 | 0.81** | 0.72* | 0.28 | -0.86 | 0.85 | 0.66** |
| MD | -0.24 | -0.36 | 0.34 | .87 | 0.28 | -0.61 | -0.36 | -0.80 | 0.23 | 0.64 | -0.37 |
| CL | 0.42 | -0.47 | 0.43 | -0.16 | 0.16 | -0.01 | 0.19 | 0.41 | -0.49 | 0.22 | 0.34 |
| CP | -0.57 | -0.56 | -0.22 | 0.41 | 0.21 | -0.69 | -0.17 | 0.31 | 0.18 | 0.35 | -0.62 |
| CS | 0.13 | 0.25 | 0.40 | 0.55 | 0.22 | -0.02 | -0.53 | -0.22 | -0.04 | 0.48 | 0.05 |
| BF | 0.17 | 0.04 | 0.52 | 0.23 | 0.74* | 0.35 | 0.11 | 0.51 | 0.12 | 0.46 | 0.31 |
| BV | 0.44 | 0.24 | 0.18 | 0.21 | 0.91* | 0.02 | 0.04 | 0.02 | 0.02 | 0.31 | 0.13 |
| k ep | 0.05 | 0.34 | 0.33 | 0.26 | 0.17 | 0.03 | 0.03 | 0.02 | 0.24 | 0.16 | 0.36 |
| PCI | 0.64 | 0.54 | 0.92 | 0.67 | 0.80 | 0.40 | 0.19 | -0.10 | 0.62 | -0.06 | 0.56 |

** correlation is significant at the 0.01 level, * Correlation is significant at 0.05 level

RA: Rheumatoid arthritis, USpA: Undifferentiated spondyloarthropathy, UCMA: undifferentiated chronic monoarthritis, OA, Osteoarthritis, FA: Fractional Anisotropy, MD: Mean Diffusivity, CL: Linear anisotropy, CP: Planar anisotropy, CS: Spherical isotropy, BV: Blood Volume, BF: Blood Flow, k ep: Volume transfer constant, PCI: post-contrast signal intensity

**Table S3. Correlation between the values of DTI and DCE –MRI indices with various cytokines in the synovial fluid of different group of arthritis patients (n=80). Results are expressed as R values**

| DTI & DCE-MRI Indices | Proinflammatory Cytokines in synovial fluid | | | | | | | | | | | | | | | | | |
|---|---|---|---|---|---|---|---|---|---|---|---|---|---|---|---|---|---|---|
| | RA (n=6) | | | Infective Arthritis (n=19) | | | USpA (n= 14) | | | UCMA (n=31) | | | OA (n=4) | | | Others (n=6) | | |
| | TNF-α | IL-1β | IL-6 | TNF-α | IL-1β | IL-6 | TNF-α | IL-1β | IL-6 | TNF-α | IL-1β | IL-6 | TNF-α | IL-1β | IL-6 | TNF-α | IL-1β | IL-6 |
| **FA** | 0.15 | 0.63** | 0.18 | 0.37* | 0.34* | 0.06 | 0.49** | 0.10 | -0.13 | 0.46** | 0.39* | 0.49* | 0.23 | 0.42** | 0.87* | 0.82* | 0.12 | 0.27 |
| **MD** | -0.61* | -0.44* | -0.96 | 0.00 | -0.01 | 0.30 | -0.56* | -0.06 | 0.02 | -0.31* | 0.36* | -0.48* | -0.57 | -0.62* | 0.54 | -0.54 | 0.38 | -0.74 |
| **CL** | 0.35 | 0.23 | -0.20 | 0.35* | 0.23 | 0.20 | 0.08 | 0.22 | 0.04 | 0.22 | 0.44* | 0.33 | 0.18 | 0.40 | 0.17 | -0.58 | 0.24 | 0.38 |
| **CP** | -0.20 | 0.00 | 0.24 | -0.20 | 0.00 | 0.24 | -0.49 | 0.20 | -0.00 | 0.30 | -0.35 | -0.33 | -0.91 | -0.31 | 0.24 | 0.38 | 0.19 | 0.91 |
| **CS** | 0.12 | -0.19 | -0.01 | 0.12 | -0.18 | 0.01 | 0.07 | 0.31 | 0.37 | -0.02 | 0.03 | 0.21 | -0.70 | 0.33 | -0.16 | -0.16 | 0.52* | 0.14 |
| **BF** | 0.12 | 0.32 | 0.21 | 0.31 | 0.11 | 0.28 | 0.07 | 0.08 | 0.03 | 0.01 | 0.08 | 0.04 | 0.02 | 0.34 | 0.32 | 0.42 | 0.32 | 0.22 |
| **BV** | 0.24 | 0.03 | 0.06 | 0.08 | 0.03 | 0.02 | 0.31 | 0.22 | 0.16 | 0.32 | 0.23 | 0.21 | 0.35 | 0.23 | 0.11 | 0.31 | 0.32 | 0.21 |
| **k ep** | 0.44 | 0.20 | 0.10 | 0.34 | 0.22 | 0.15 | 0.17 | 0.01 | 0.17 | 0.19 | 0.05 | 0.04 | 0.23 | 0.03 | 0.44 | 0.16 | 0.34 | 0.27 |
| **PCI** | 0.03 | 0.42 | 0.14 | 0.03 | 0.42 | 0.14 | -0.25 | 0.26 | 0.07 | 0.32 | -0.03 | -0.15 | 0.31 | 0.57* | 0.27 | 0.22 | 0.54 | 0.14 |

** correlation is significant at the 0.01 level, * Correlation is significant at 0.05 level

RA: Rheumatoid arthritis, USpA: Undifferentiated spondyloarthropathy, UCMA: undifferentiated chronic monoarthritis, OA, Osteoarthritis, FA: Fractional Anisotropy, MD: Mean Diffusivity, CL: Linear anisotropy, CP: Planar anisotropy, CS: Spherical isotropy, BV: Blood Volume, BF: Blood Flow, k ep: Volume transfer constant, PCI: post-contrast signal intensity

**Table S4 : Multivariate linear regression for predicting Total cells infiltrating the synovium as dependent variable and DTI and DCE metrics as independent variables**

| Model | | Unstandardized Coefficients B | Unstandardized Coefficients Std. Error | Standardized Coefficients Beta | t | Sig. | R | R Square | Std error of the estimate | Sig. F Change |
|---|---|---|---|---|---|---|---|---|---|---|
| 1 | (Constant) | -203.454 | 35.285 | | -5.766 | .000 | 0.915 | 0.836 | 44.06 | 5.31E-32 |
| | FA | 3138.043 | 158.157 | .915 | 19.841 | 5.31E-32 | | | | |
| 2 | (Constant) | -600.417 | 171.327 | | -3.505 | .001 | 0.921 | 0.848 | 42.81 | 0.021 |
| | FA | 3277.215 | 164.528 | .955 | 19.919 | 6.8E-32 | | | | |
| | CS | 486.244 | 205.617 | .113 | 2.365 | .021 | | | | |
| 3 | (Constant) | -642.382 | 167.546 | | -3.834 | .000 | 0.926 | 0.858 | 41.62 | 0.023 |
| | FA | 3101.497 | 176.925 | .904 | 17.530 | 3.04E-28 | | | | |
| | CS | 550.851 | 201.832 | .128 | 2.729 | .008 | | | | |
| | KEP | 13.143 | 5.654 | .117 | 2.324 | .023 | | | | |

FA; Fractional anisotropy, CS; Spherical isotropy, Kep; Volume transfer constant

**Table S5: Multivariate linear regression for predicting CD34 as dependent variable and DTI and DCE metrics as independent variables**

| Model | | Unstandardized Coefficients | | Standardized Coefficients | t | Sig. | R | R Square | Std error of the estimate | Sig. F change |
|---|---|---|---|---|---|---|---|---|---|---|
| | | B | Std. Error | Beta | | | | | | |
| 1 | (Constant) | 19.185 | 3.431 | | 5.592 | .000 | 0.765 | 0.585 | 10.82 | 2.32E-16 |
| | BF | .309 | .030 | .765 | 10.418 | 2.32E-16 | | | | |
| 2 | (Constant) | 17.073 | 3.155 | | 5.412 | .000 | 0.814 | 0.662 | 9.82 | 0.000078 |
| | BF | .180 | .041 | .444 | 4.371 | 0.000039 | | | | |
| | BV | 1.735 | .415 | .425 | 4.178 | 0.000078 | | | | |

BF; Blood flow, BV; Blood volume
